# The Role of CTGF in Liver Fibrosis Induced in 3D Human Liver Spheroids

**DOI:** 10.3390/cells12020302

**Published:** 2023-01-13

**Authors:** Sara Redenšek Trampuž, Sander van Riet, Åsa Nordling, Magnus Ingelman-Sundberg

**Affiliations:** 1 Section of Pharmacogenetics, Department of Physiology and Pharmacology, Karolinska Institutet, SE-171 77 Stockholm, Sweden; 2 Pharmacogenetics Laboratory, Institute of Biochemistry and Molecular Genetics, Faculty of Medicine, University of Ljubljana, Vrazov trg 2, 1000 Ljubljana, Slovenia

**Keywords:** NASH, NAFLD, hepatitis, angiotensin II, metabolic syndrome, PNPLA3

## Abstract

Connective tissue growth factor (CTGF) is involved in the regulation of extracellular matrix (ECM) production. Elevated levels of CTGF can be found in plasma from patients with liver fibrosis and in experimental animal models of liver fibrosis, but the exact role of CTGF in, e.g., diet-induced human liver fibrosis is not entirely known. To address this question, we utilized a 3D human liver co-culture spheroid model composed of hepatocytes and non-parenchymal cells, in which fibrosis is induced by TGF-β1, CTGF or free fatty acids (FFA). Treatment of the spheroids with TGF-β1 or FFA increased COL1A1 deposition as well as the expression of TGF-β1 and CTGF. Recombinant CTGF, as well as angiotensin II, caused increased expression and/or production of CTGF, TGF-β1, COL1A1, LOX, and IL-6. In addition, silencing of CTGF reduced both TGF-β1- and FFA-induced COL1A1 deposition. Furthermore, we found that IL-6 induced CTGF, COL1A1 and TGF-β1 production, suggesting that IL-6 is a mediator in the pathway of CTGF-induced fibrosis. Taken together, our data indicate a specific role for CTGF and CTGF downstream signaling pathways for the development of liver inflammation and fibrosis in the human 3D liver spheroid model.

## 1. Introduction

Non-alcoholic fatty liver disease (NAFLD) is one of the most common liver diseases worldwide, with a prevalence of about 25% in the general population [1]. Subjects with NAFLD are at high risk of developing non-alcoholic steatohepatitis (NASH), characterized by inflammation and fibrosis of the liver [2]. There are several risk factors for development of NAFLD, such as high-fat diet, obesity, diabetes, age and also genetic polymorphisms, of which *PNPLA3* (rs738409) and *TM6SF2* (rs58542926) are the most important [2]. The cellular mechanisms and signaling cascades of this process are still largely unknown and no effective treatments or targeted therapeutics are available [3]. To a great extent, this might be due to the absence of versatile human liver NASH models since information hitherto obtained based on animal models or inappropriate in vitro models has not provided a basis for the development of successful anti-NASH drugs. Hepatic fibrogenesis is a dynamic wound-healing process occurring during chronic injury of the liver parenchyma that can result in excess deposition of extracellular matrix (ECM) components, leading to scar formation in the extracellular space of the liver parenchyma, displacing hepatocytes [4,5,6]. Although this process involves different liver cell types, the ECM is predominantly produced by the activated hepatic stellate cells (HSC) in response to different stimuli such as toxins, viruses, oxidative stress and inflammation [6,7,8].

Connective tissue growth factor (CTGF) is a matricellular protein of the CCN family that is involved in the regulation of ECM production [9]. It is an ECM-associated heparin-binding protein able to interact with several receptors, growth factors and signaling molecules [4]. However, no specific primary target/receptor for CTGF has yet been identified, although it binds to various cell surface proteins in a context-dependent manner, such as integrins, ECM components and various receptors [4,10,11]. CTGF has been shown to be involved in different cellular processes, such as proliferation, differentiation, migration, adhesion, ECM synthesis and interaction with matrix components [4,7,9,11,12]. The cellular distribution of CTGF in the liver depends on the status of health or disease etiology. CTGF is mainly produced by HSC, however, hepatocytes, portal fibroblasts and cholangiocytes are also capable of producing CTGF [4,13]. Elevated levels of CTGF can be found in plasma from patients with liver fibrosis and in experimental animal models of liver fibrosis [12,13].

TGF-β1 is a central cytokine in the fibrotic process and the main driver of HSC activation and HSC-mediated ECM production following metabolic stress [14]. It signals via both the canonical and non-canonical Smad pathways [15], such as mitogen-activated protein kinase pathways mediated by extracellular-signal-regulated kinase (ERK), p38 and JUN *N*-terminal kinase (JNK) as well as RHO-associated kinase (ROCK), RAC-α serine/threonine protein kinase (AKT) and Hippo pathways [8,16,17]. All these pathways are involved in processes resulting in ECM deposition and the release of regulatory proteins, such as CTGF. In turn, CTGF is able to regulate TGF-β1 production and further activate HSC in a mouse model, creating a positive feedback loop [18]. During an uncontrolled activation of this loop caused by chronic inflammation of the liver, large amounts of ECM proteins, such as collagens and integrins, are deposited [8,18].

We have previously developed a human in vitro 3D liver co-culture model of FFA-induced liver fibrosis [19]. These 3D liver co-culture spheroids contain primary human hepatocytes (PHH) and non-parenchymal cells (NPC) and display a phenotype similar to the in vivo human liver, as evident from transcriptomic, proteomic and metabolomic profiles [20,21,22]. This model allows for monitoring of long term changes following different stimuli, as well as the role of different cell types and specific genes, based on the ability to perform gene specific silencing. In the current investigation we applied this model to identify the role of CTGF function in liver fibrosis. The results demonstrate a significant contribution of CTGF to enhanced fibrotic pathology in response to fatty acids and other pro-fibrotic stimuli.

## 2. Materials and Methods

### 2.1. Spheroid Cultures

Cryopreserved PHH and NPC were obtained from KaLy-Cell (KLC; Plobsheim, France) and Lonza (Basel, Switzerland). NPC were passaged as previously described [23]. The PHH and NPC donor characteristics are listed in Table 1. PHH and NPC were seeded as previously described [20,23]. Briefly, PHH were seeded in 96-well Corning Costar Ultra-Low Attachment Plates (Merck, Kenilworth, NY, USA) or in 96-Well Nunclon Sphera U-Shaped-Bottom Microplate plates (Thermo Fisher, Waltham, MA, USA) with NPC at a 4:1 (1500:375) ratio. Spheroids were cultured in William’s E medium supplemented with 2 mM L-glutamine (Sigma-Aldrich, Saint Louis, MO, USA), 100 units/mL penicillin (Sigma-Aldrich), 100 μg/mL streptomycin (Sigma-Aldrich), 100 nM dexamethasone (Sigma-Aldrich), ITS X-100 (Thermo Fisher) and 10% fetal bovine serum (FBS; Thermo Fisher). At day 5 after seeding, the spheroids were refreshed with the medium as described above without the addition of FBS, and afterwards, every 2 to 3 days. Induction of liver fibrosis and treatment of spheroids with different compounds was initiated on day 7 after seeding. Spheroids were treated for either 3 or 7 days and harvested at day 10 or 14 after seeding. Spheroids were cultured in 100 μL of medium under standard cell culture conditions at 37 °C in a humidified incubator at 5% CO_2_. The combination of donor 1 PHH and donor 3 NPC was used, unless stated otherwise. The term monocultures refers to spheroids comprised of PHH only, while the term co-cultures refers to spheroids comprised of both PHH and NPC.

### 2.2. Functional Responses

**Figure 1:**
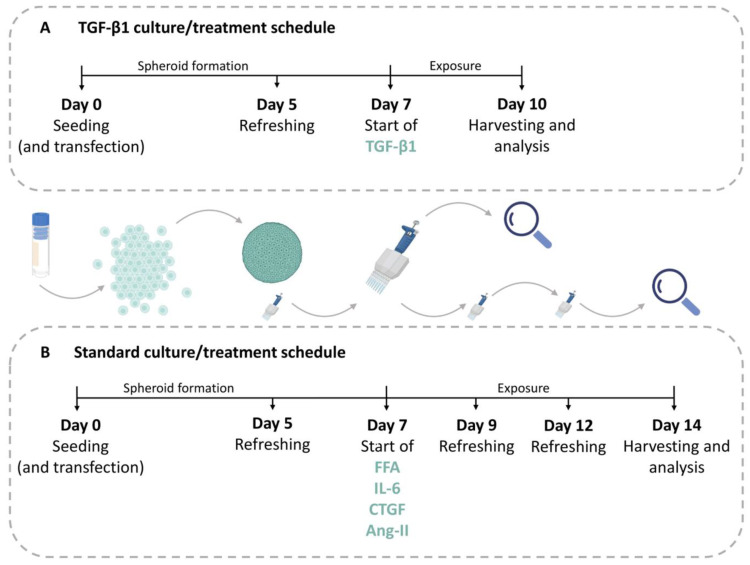
Schematic overview of experimental setup. (**A**) For analyses of effects of TGF-β1 treatment the cells were seeded (day 0) and cultivated to form spheroids for 5 days when the first medium refreshment occurred. TGF-β1 treatment started on day 7 and lasted for 3 days, after which the spheroids were harvested. When required, siRNA and transfection reagents were added during seeding at day 0 to achieve a high transfection efficiency. Following transfection, the cells were treated as above. (**B**) In the standard treatment schedule, the cells were seeded on day 0 and the first medium refreshment took place on day 5. All treatments of FFA, IL-6, CTGF, or angiotensin II (Ang-II), started on day 7 and lasted for 7 days. Medium and compounds were refreshed every 2 to 3 days, until the spheroids were harvested. In experiments, where cells were transfected, this took place on day 0 along with the seeding procedure. TGF-β1—transforming growth factor beta 1; FFA—free-fatty acids; IL-6—interleukin 6; CTGF—connective tissue growth factor; Ang-II—angiotensin II. Parts of the figure were created with BioRender.com.

To activate the TGF-β1 signaling, spheroids were treated with recombinant TGF-β1 (R&D systems, Minneapolis, MN, USA) (5 ng/mL), dissolved in a vehicle as per manufacturer’s instructions, for 3 days, as previously described, starting on day 7 after seeding [19] (Figure 1A). For induction of a NASH-like phenotype, spheroids were treated with a lipogenic cocktail comprised of palmitic and oleic acid, as previously described [24], with minor modifications. Unsaturated oleic acid and saturated palmitic acid (Sigma Aldrich), solubilized in ethanol, were bound to 10% bovine serum albumin at a 1:5 molar ratio for 2 h at 40 °C. The free fatty acids (FFA) were combined in a 1:1 ratio and spheroids were exposed to 240 µM palmitic acid and 240 µM oleic acid from day 7 to day 14, with medium refreshed every 2 to 3 days (Figure 1B). To assess effects of IL-6, spheroids were treated with the recombinant IL-6 (Thermo Fisher) (1.5 ng/mL), dissolved in a vehicle, as per manufacturer’s instructions, from day 7 to day 14 (Figure 1B). To study the CTGF downstream signaling, spheroids were treated with the recombinant CTGF (Thermo Fisher) (100 ng/mL), dissolved in a vehicle, as per manufacturer’s instructions, from day 7 to day 14 as well [25] (Figure 1B). To pharmacologically induce CTGF production, spheroids were treated with angiotensin II (Sigma-Aldrich), dissolved in water, at different concentrations, (10^−5^ M, 10^−6^ M, and 10^−7^ M) between days 7 and 14 [26] (Figure 1B). Vehicles used for dissolving the compounds were used as corresponding control conditions in all of the experiments.

### 2.3. siRNA-Mediated CTGF Silencing

Transfection with small interfering RNA (siRNA) was performed on the day of seeding according to the manufacturer’s instructions with a few modifications. The siRNA (CTGF siRNA, Silencer Select, Thermo Fisher) and its scrambled control (Thermo Fisher) were mixed with OptiMEM (Thermo Fisher) to a concentration of 50 nM. Additionally, lipofectamine RNAiMAX (Thermo Fisher) was mixed with OptiMEM to obtain a final volume of 0.2 μL of Lipofectamine RNAiMAX per well. Following a 5 min incubation of both mixtures, they were carefully, in a dropwise manner, mixed at a 1:1 ratio. Subsequently, the mixture was incubated at room temperature for 20 min. The transfection siRNA–lipid complex solution was mixed with the cell suspension. Cells were then seeded and maintained as described above (Figure 1).

### 2.4. Cell Viability

The ATP level was measured on the day of spheroid harvesting using at least 4 different spheroids per condition. CellTiter Glo Luminescent Cell Viability Assay kit (Promega, Madison, WI, USA) was used according to manufacturer’s instructions. First, 80 μL of the 100 μL medium was removed from the wells and 25 μL of reconstituted assay reagent was added per well. Spheroids were mechanically disrupted by pipetting and the plate was incubated for 20 min in the dark at 37 °C. The luminescence signal was measured using a MicroBeta LumiJET 2460 Microplate Counter (Perkin Elmer, Waltham, MA, USA) in white flat bottom 96-well plates.

### 2.5. RNA Isolation and cDNA Synthesis

Total RNA isolation was performed using QIAzol lysis reagent (Qiagen, Hilden, Germany) using 48 spheroids. After the lysis, chloroform was added and the mixture was thoroughly mixed and centrifuged. The aqueous phase was combined with the same volume of isopropanol. After the centrifugation, the supernatant was discarded and the pellet was washed with 70% ethanol at least twice. The dried pellet was resuspended in ddH_2_O. RNA concentration was determined using Qubit 4 Fluorometer (Thermo Fisher). Up to 500 ng of the total RNA was reverse transcribed into cDNA with SuperScript III reverse transcriptase (Thermo Fisher) using a SimpliAmp Thermal Cycler (Thermo Fisher) according to the manufacturer’s protocol. The transcribed cDNA was stored at −20 °C until further use.

### 2.6. Gene Expression Analysis

Amplification reactions were performed using a 2X TaqMan Universal PCR mix (Thermo Fisher) on a 7500 Fast Real-Time PCR system (Applied Biosystems, Waltham, MA, USA) with 20X TaqMan probes (Table S1). Gene expression was analyzed using the delta-delta Ct method (2^−ΔΔCt^) using *TBP* as the reference gene and normalized to the respective control.

### 2.7. Immunohistochemistry

Spheroids were fixed for 2 h at room temperature in 4% paraformaldehyde and subsequently dehydrated in 30% sucrose for at least 2 days at 4 °C. The spheroids were embedded and frozen using Tissue-Tek OCT (Sakura Finetek, Alphen aan den Rijn, The Netherlands) and sectioned at 8 μm using a NX70 Cryostat (Thermo Fisher). Sections of spheroids were blocked with 5% BSA, 0.25% Triton X-100 in PBS (Phosphate-buffered saline) (PBS/BSA/Triton, Sigma Aldrich) for 2 h at room temperature. A list of primary and secondary antibodies can be found in Table S2.

Antibodies were diluted in PBS/BSA/Triton. Sections of spheroids were stained with the primary antibody overnight at 4 °C. Afterwards, slides were washed with PBS 3 times for 15 min. Secondary antibodies were diluted in PBS/BSA/Triton as well. Secondary antibody staining lasted for 2 h at room temperature in the dark. Afterwards, slides were washed with PBS 3 times for 15 min. Slides were mounted with ProLong Gold Antifade Mountant with DAPI (Thermo Fisher), imaged using the Olympus IX73 inverted microscope (Olympus, Tokyo, Japan) and processed using StreamView software version 1.9.4. At least ten 8 μm sections of different spheroids were imaged per condition per experiment. For quantification, the integrated density of the respective staining was divided by the area of the DAPI staining, which was named as fluorescence intensity in the plots. Measurements were obtained using Image J software. Every dot in the plots presents the average fluorescence intensity of at least 10 imaged sections of different spheroids per condition per experiment. The size of the spheroids is marked with a scale bar of 100 μm.

### 2.8. Immunohistochemistry Staining of 2D Cultured Non-Parenchymal Cells

A round cover slip was introduced into the 12 wells of the plate. NPC were thawed and added to the wells using the same medium as described for the spheroids above. After the cells had attached, which usually took 2 days, the medium was removed, cells were washed with PBS, and fixed in 4% paraformaldehyde for 15 min. Immunohistochemistry (IHC) staining was performed as described above. Cover slips were mounted with ProLong Gold Antifade Mountant with DAPI (Thermo Fisher) on a glass slide, imaged using the Olympus IX73 inverted microscope (Olympus, Tokyo, Japan) and processed using StreamView software version 1.9.4. Ten images per staining were produced. For the purpose of NPC characterization, the quantification was performed as followed. All nuclei were counted across all 10 images, followed by counting only the cells positive for the respective staining across all 10 images. The percentage of positive cells for the respective staining is presented in the plot.

### 2.9. ELISA

The Human Pro-Collagen I α1 DuoSet ELISA kit (R&D systems) and the Human CTGF/CCN2 DuoSet ELISA kit (R&D systems) were used according to manufacturer’s protocol to determine pro-collagen I α1 and CTGF concentrations in the culture medium, respectively. The medium samples were centrifuged immediately after harvesting at 2000× *g* for 10 min to remove debris and the supernatant was stored at −20 °C until further use.

### 2.10. Statistical Analysis

Quantitative data were analyzed using GraphPad Prism version 5 (GraphPad Software, San Diego, CA, USA) and described as the mean and standard error of the mean (SEM). Statistical analysis of differences induced by stimuli was carried out using a paired, two-sided *t*-test and comparing each condition to control.

### 2.11. Ethical Approval

PHH and NPC were obtained from commercially available sources and required no ethical approval by Karolinska Institutet. Ethical approval for the donors, received from the project partners at KaLy Cell, was obtained. Copies of documentation from Lonza and BioIVT regarding written consent by donors was obtained from the respective companies.

## 3. Results

### 3.1. TGF-β1 Induces ECM Deposition and Increases CTGF Production in 3D Human Liver Spheroids

**Figure 2:**
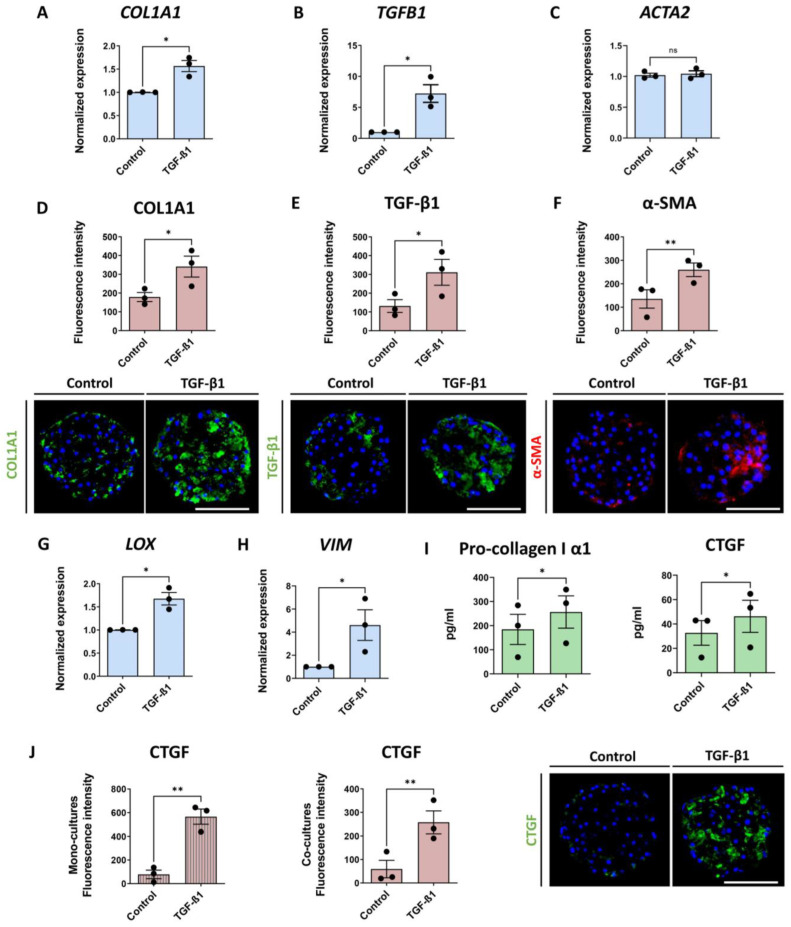
TGF-β1 treatment of 3D human liver spheroids increases fibrotic markers as well as CTGF. Three-dimensional human liver spheroids were treated with 5 ng/mL of TGF-β1 for 3 days. Blue bars represent mRNA expression data. Red bars show results of IHC analysis, where each dot represents an average result of at least 10 analyzed sections of spheroids in 1 separate experiment. Lastly, green bars represent results of ELISA analyses. (**A**–**C**,**G**,**H**) Transcription of *COL1A1*, *TGFB1*, *LOX* and *VIM* was increased 3 days after treatment initiation, while *ACTA2* expression was unaffected (n = 3). (**D**–**F**) Representative images of key fibrosis related markers at day 10 including quantification of the increase of COL1A1, TGF-β1 and α-SMA expression are shown (n = 3). (**I**) TGF-β1 treatment increased the pro-collagen I α1 and CTGF released into the culture medium. (**J**) CTGF was increased in both monoculture (**left**) and co-culture (**middle**) spheroids in response to TGF-β1 treatment (n = 3), which is also visualized by immunofluorescence (**right**). In the Figure 2, all of the data presented, with the exception of (**J**) ((**left**), monocultures), represents experiments performed in co-culture spheroids. Nuclei in the IHC images are shown in blue. The size of the spheroids is marked with a white scale bar of 100 μm. Data are shown as mean ± SEM. Statistical analysis—Student’s *t*-test; * *p* < 0.05; ** *p* < 0.01; ns: not significant.

In order to determine whether the liver co-culture spheroids could produce CTGF, they were treated with 5 ng/mL of TGF-β1 from day 7 until day 10 and analyzed for fibrotic markers and CTGF production (Figure 1A). We chose TGF-β1 due to its high profibrotic potential. The co-culture spheroids consisted of PHH and NPC, which were predominantly comprised of HSC, as shown in Figure S1A. Treatment with TGF-β1 caused an increased mRNA expression of *COL1A1*, *TGFB1*, *LOX* and *VIM*, but not of *ACTA2* (Figure 2A–C,G,H). A comparable increase was detected at the protein level for COL1A1, TGF-β1 and also α-SMA (Figure 2D–F). The response of COL1A1 deposition in the spheroids subsequent to TGF-β1 treatment was rather variable among different spheroids, which we took into account by the analysis of at least 10 spheroids in every experiment for every condition (see an example in Figure S1B). An average increase in COL1A1 deposition was supported by the increase of pro-collagen I α1, a precursor of mature COL1A1, in the culture medium (Figure 2I) over the 3 days of treatment. An increase in CTGF production was observed upon treatment with TGF-β1 in the culture medium and also by IHC (Figure 2I,J). CTGF production was greatly elevated after TGF-β1 treatment in both mono- (PHH only) and co-cultures (PHH combined with NPC) (Figure 2J), also showing a variable response among spheroids (see an example in Figure S1B).

When culturing NPC in 2D, we observed autonomous CTGF production. However, we observed no CTGF production in freshly thawed, unstimulated PHH (Figure S1C). This indicates that PHH can produce CTGF upon TGF-β1 stimulation, as previously shown [27], but does not exclude the possibility of TGF-β1-induced phenotypic changes in the cells, which might also increase CTGF production. The latter is supported by the increased deposition of COL1A1, and TGF-β1 in monoculture spheroids upon TGF-β1 treatment, whereas α-SMA expression was not increased. (Figure S1D). Since TGF-β1 affected the spheroid viability even after a short 3-day treatment (Figure S1E), we also investigated FFA as a more relevant and less toxic inducer of fibrosis.

### 3.2. Both Free Fatty Acids and IL-6 Increase CTGF Production and Fibrotic Markers

**Figure 3:**
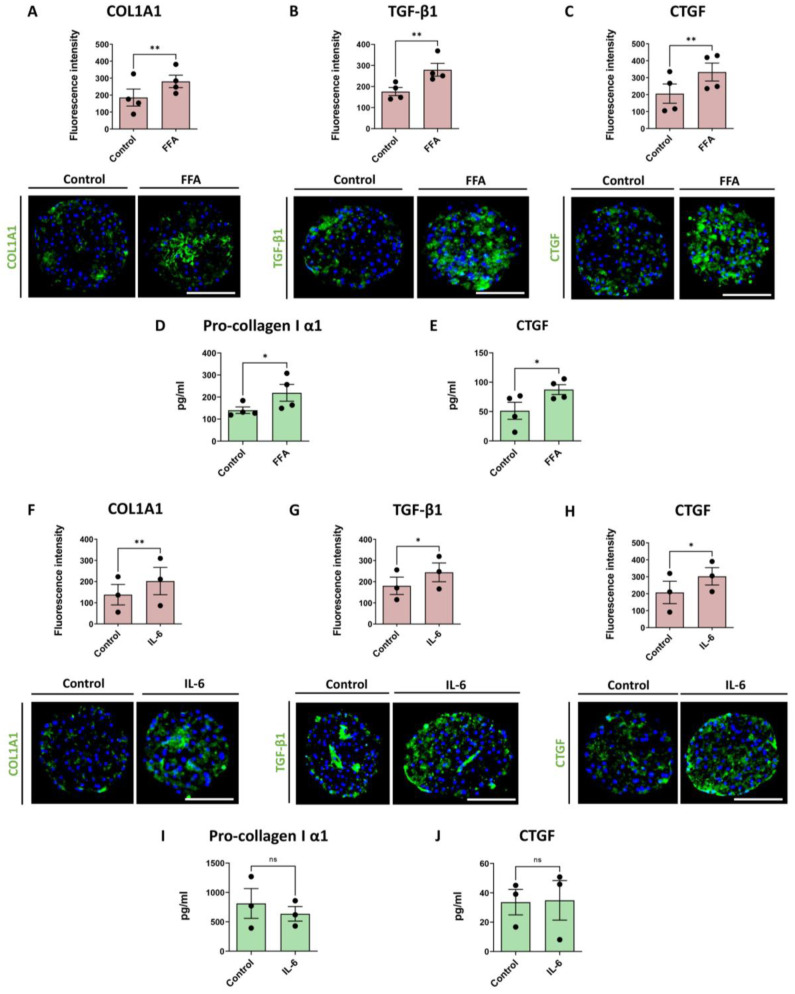
FFA and IL-6 treatment increase fibrosis markers and CTGF production in co-cultures. Red bars represent results of IHC analysis and green bars represent data based on ELISA analyses. (**A**–**C**) Treatment of co-culture spheroids with FFA increased production of COL1A1, TGF-β1 and CTGF in the spheroids (n = 4). (**D**,**E**) FFA treatment increased pro-collagen I α1 and CTGF released from the cells into the medium during the last 2 days of culture (n = 4). (**F**–**H**) Treatment of the spheroids with 1.5 ng/mL of IL-6 for 7 days increased production of COL1A1, TGF-β1 and CTGF (n = 3), (**I**,**J**) but not of pro-collagen I α1 and CTGF production monitored by ELISA (n = 3). Every dot in the plots represents data from 1 experiment. Every dot in the red bars represents an average of at least 10 analyzed sections of spheroids per experiment. The size of the spheroids is marked with a white scale bar of 100 μm. Data are shown as mean ± SEM. Statistical analysis—Student’s *t*-test; * *p* < 0.05; ** *p* < 0.01; ns: not significant.

We have previously shown that treatment with FFA mimics the Western diet and can induce steatosis, HSC activation and collagen deposition [19]. Here, we evaluated FFA-induced COL1A1 deposition as well as TGF-β1 production in the liver spheroids, as described in Figure 1B. We confirmed the ability of the present model to develop a fibrotic phenotype, as revealed by increased COL1A1 and TGF-β1 expression (Figure 3A,B) and by the accumulation of pro-collagen I α1 in the culture medium during the last 2 days of culture (Figure 3D). Similarly, we observed an elevated CTGF production within spheroids after FFA treatment (Figure 3C) and increased CTGF concentration in the culture medium, measured at day 14 (Figure 3E).

IL-6 is one of the main pro-inflammatory mediators with elevated concentrations in blood plasma in NAFLD patients [28]. We evaluated the effect of a 7-day IL-6 treatment (1.5 ng/mL) on co-culture spheroids (Figure 1B). We found elevated COL1A1 levels (Figure 3F) and increased TGF-β1 and CTGF deposition in the spheroids (Figure 3G,H), whereas no increased CTGF concentration or secreted pro-collagen I α1 was seen in the culture medium (Figure 3I,J).

### 3.3. Treatment with CTGF Increases Production of Fibrotic Markers

**Figure 4:**
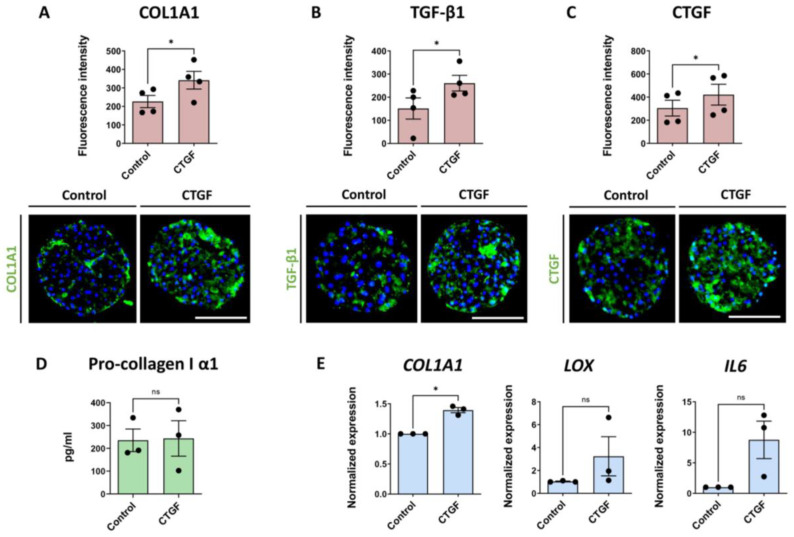
CTGF contributes to liver fibrosis in the 3D liver co-culture fibrosis model. Blue bars represent mRNA expression data. Red bars represent results of IHC analysis and green bars represent data based on ELISA analyses. (**A**–**C**) CTGF treatment significantly increased the production of COL1A1, TGF-β1 and CTGF, as revealed by IHC (n = 4); (3 experiments with PHH donor 1 and 1 experiment with PHH donor 2; NPC from donor 3 were used in all 4 experiments). (**D**) Pro-collagen I α1 release was not affected by the CTGF treatment (n = 3) (**E**) *COL1A1*, *LOX* and *IL6* mRNA levels were increased after a 7-day CTGF treatment, although the changes in *LOX* and *IL6* expression did not reach statistical significance (n = 3, 2 experiments with the PHH donor 1 and 1 experiment with PHH donor 2; NPC from donor 3 in all 3 experiments). Every dot represents data from 1 experiment. Every dot in the red bars represents an average of at least 10 analyzed sections of spheroids per experiment. The size of the spheroids is marked with a white scale bar of 100 μm. Data are shown as mean ± SEM. Statistical analysis—Student’s *t*-test; * *p* < 0.05; ns: not significant.

As described above, elevated CTGF production was induced by TGF-β1, FFA and IL-6. Hence, we also wanted to evaluate the effects of exogenous CTGF on liver spheroids according to the experimental layout presented in Figure 1B. Treatment with recombinant CTGF showed no change in ATP and albumin levels, indicating no loss in viability and functionality (Figure S2). After a 7-day treatment, a significant increase in COL1A1 production was observed (Figure 4A), but the increase was lower compared to TGF-β1- or FFA-induced COL1A1 deposition. Interestingly, TGF-β1 production was also significantly increased by CTGF treatment (Figure 4B), as well as CTGF itself (Figure 4C). CTGF staining shows a specific signal throughout the spheroid, including the core, indicating that this is endogenous CTGF production. We also determined the concentration of pro-collagen I α1 release into the medium in the last 2 days of culture, but did not observe any significant differences (Figure 4D). The difference in the *COL1A1* mRNA levels were comparable with the IHC data. We also observed increases of *LOX* and *IL6* mRNA, however, without statistical significance (Figure 4E).

### 3.4. Pharmacological Induction of CTGF Synthesis Contributes to Fibrosis Development

**Figure 5:**
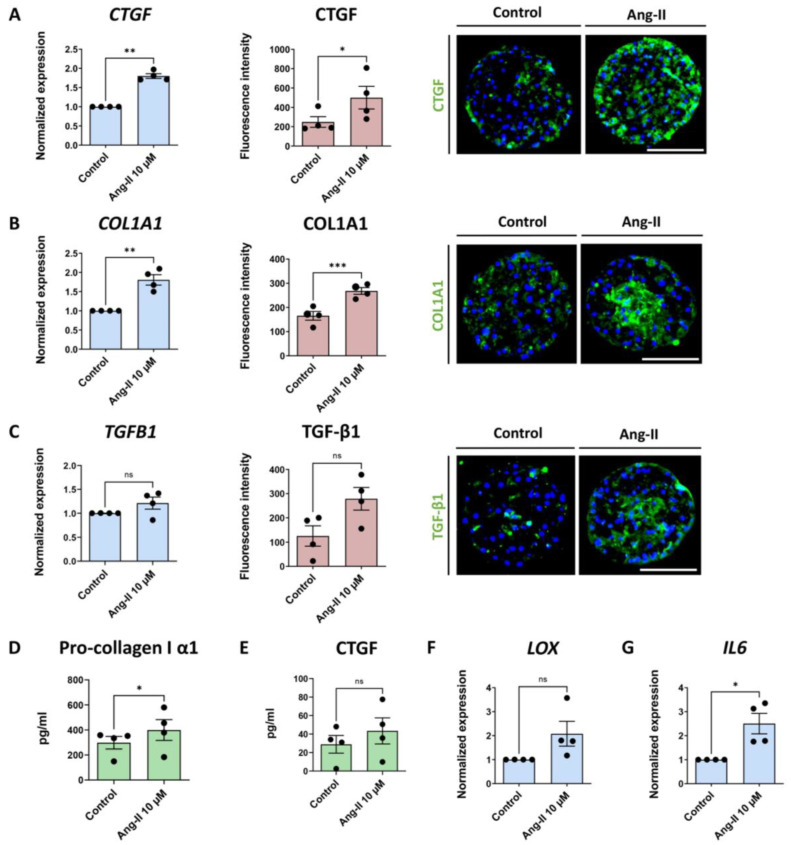
Angiotensin II induces CTGF production and markers of liver fibrosis. Angiotensin II was used to increase CTGF production and induce fibrosis in liver spheroids. Blue bars present the mRNA level data, red bars, results of IHC analysis, and green bars, data acquired using ELISA. (**A**) Angiotensin II caused an increase in CTGF expression (n = 4; 3 experiments with the PHH donor 1 and 1 experiment with PHH donor 2; NPC from donor 3 in all 4 experiments) and production (n = 4; 3 experiments with the PHH donor 1 and 1 experiment with PHH donor 2; NPC from donor 3 in all 4 experiments). (**B**) Expression and protein production of COL1A1 increased after angiotensin II treatment (n = 4). (**C**) TGF-β1 was increased on the protein level (n = 4), however the transcriptional level was unaffected (n = 4). (**D**) Pro-collagen I α1 released from the cells increased during the last 2 days of culture of the angiotensin II-treated spheroids (n = 4). (**E**) Similarly, the CTGF concentration in the culture medium was increased in all 4 experiments, although not to a significant extent (n = 4). (**F**,**G**) *LOX* and *IL6* mRNA expression increased due to angiotensin II treatment (n = 4; 3 experiments with PHH donor 1 and 1 experiment with PHH donor 2; NPC from donor 3 in all 4 experiments). Nuclei in the IHC images are shown in blue. Every dot in the plots represents data from 1 experiment. Every dot in the red graphs is the average of at least 10 analyzed sections of spheroids per experiment. The size of the spheroids is marked with a white scale bar of 100 μm. Data are shown as mean ± SEM. Statistical analysis—Student’s *t*-test; * *p* < 0.05; ** *p* < 0.01; *** *p* < 0.001; ns: not significant. Ang-II—angiotensin II.

To further understand the role of CTGF in the progression of fibrosis, we sought to increase the CTGF deposition by a pharmacological intervention. Treatment with angiotensin II has been described to induce CTGF production via stimulation of the AT1 receptor in human LX-2 cells. The subsequent downstream signaling activated the NF-κB and Smad2/3 cross-talk pathway, by which COL1A1 was induced [26]. We applied this approach to our 3D liver spheroid model. The spheroids were treated for 7 days with different concentrations of angiotensin II, according to the scheme in Figure 1B, and a dose-dependent increase of CTGF both at the transcript and protein levels was observed (Figure S3). We subsequently used the highest tested concentration of angiotensin II (10 μM). This treatment caused significantly increased CTGF and COL1A1 expression, both at the mRNA and protein levels (Figure 5A,B), as well as of TGF-β1 protein at day 14 (Figure 5C). Monitoring pro-collagen I α1 release in the medium during the last 2 days of culture revealed a significant increase (Figure 5D), whereas CTGF concentration in the culture medium was not increased significantly (Figure 5E). Furthermore, an elevation in *LOX* and *IL6* expression was detected (Figure 5F,G). Taken together, the data indicate that angiotensin II increases production of CTGF in the liver spheroids, which relates to increased COL1A1 expression.

### 3.5. CTGF Silencing Decreases COL1A1 Production Induced by TGF-β1

**Figure 6:**
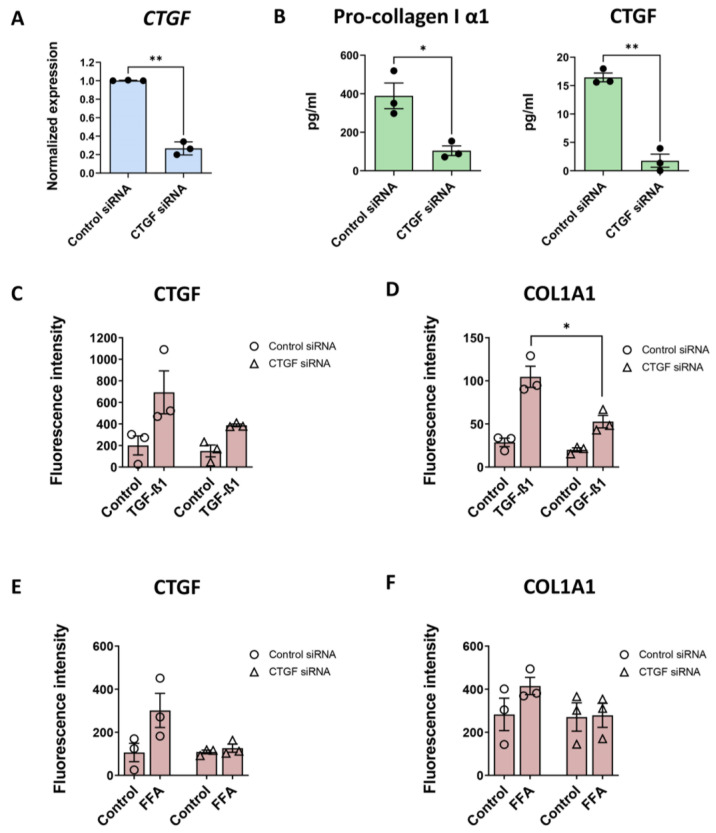
CTGF silencing reduces the COL1A1 production induced by TGF-β1. CTGF silencing was evaluated for TGF-β1- and FFA-induced CTGF and COL1A1 deposition. Spheroids were maintained and treated as shown in Figure 1. Blue bars present the mRNA level data, red bars present results of IHC analysis and green bars, data acquired using ELISA. (**A**) Silencing of CTGF was confirmed at the transcriptional level 10 days after seeding and transfection (n = 3). (**B**) CTGF silencing also reduced baseline pro-collagen I α1 and CTGF released in the medium in the last 3 days of culture (n = 3). (**C**,**D**) CTGF siRNA-transfection caused a decrease in TGF-β1-induced CTGF and COL1A1 production (n = 3). TGF-β1-induced COL1A1 production was significantly lower in the spheroids with silenced CTGF. (**E**,**F**) CTGF siRNA-transfection also caused a decrease in FFA-induced CTGF and COL1A1 production, although the difference was not significant. Every dot in the graphs represents data from 1 experiment and those in the red bars present an average of at least 10 analyzed sections of spheroids per experiment. Data are shown as mean ± SEM. Statistical analysis—Student’s *t*-test; * *p* < 0.05; ** *p* < 0.01.

The role of CTGF in the fibrotic model was also investigated by gene silencing. Following transfection with CTGF siRNA, *CTGF* mRNA expression was reduced by ~70% compared to control siRNA (Figure 6A). CTGF silencing significantly decreased baseline pro-collagen I α1 production in the medium, as well as CTGF production (Figure 6B). The TGF-β1-induced CTGF production was largely suppressed after CTGF silencing (Figure 6C). A similar impaired production of COL1A1 was observed after TGF-β1 treatment (Figure 6D). In accordance, although less pronounced, was the reduced increase of CTGF due to FFA treatment after CTGF silencing (Figure 6E). The latter translated into COL1A1 production even upon FFA treatment as well (Figure 6F). CTGF silencing in the FFA-induced fibrosis caused a relatively less pronounced decrease in COL1A1 production than observed in TGF-β1-induced fibrosis, indicating a higher contribution of CTGF-independent signal transduction processes for development of fibrosis after FFA treatment.

## 4. Discussion

**Figure 7:**
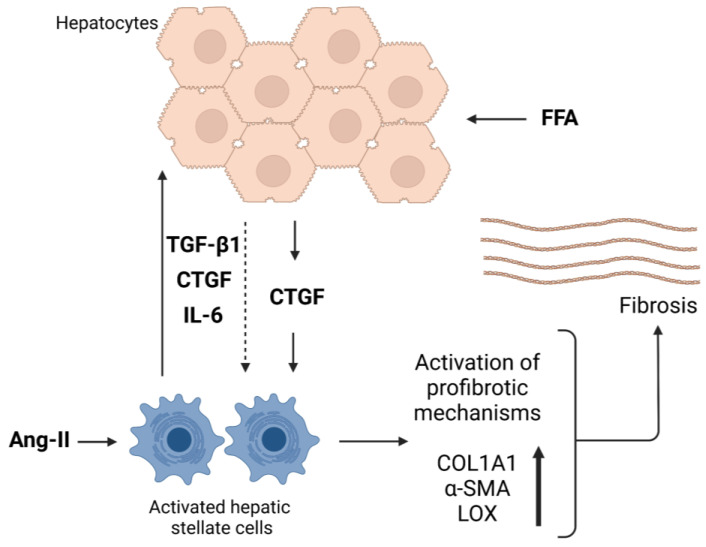
Proposed schematic overview of CTGF’s function in liver fibrosis in the spheroid model. Under stress conditions, CTGF can be produced by both hepatocytes and activated hepatic stellate cells and can, in addition, itself induce production and release of TGF-β1, IL-6 and CTGF from the co-culture spheroids. Free fatty acids (FFA) create a profibrotic environment, in which co-culture spheroids produce CTGF. Consequently, profibrotic mediators are released from hepatic stellate cells. The profibrotic mechanisms are activated and the production of COL1A1, α-SMA and LOX increases. ECM then accumulates in the spheroids as a final consequence of FFA treatment. Angiotensin II binds to the AT1 receptor on hepatic stellate cells and, through its downstream signaling, induces the production of CTGF and, consequently, other mediators as well. α-SMA—α smooth muscle actin; Ang-II—angiotensin II; COL1A1—collagen, type I, α1; CTGF—connective tissue growth factor; FFA—free fatty acids; IL-6—interleukin 6; LOX—lysyl oxidase; TGF-β1—transforming growth factor beta 1. Created with BioRender.com.

To date, the role of CTGF in liver fibrotic events has been mainly studied in immortalized cell lines and murine models, but experiments in primary human models are lacking. Here we present CTGF data, confirming its supportive role in liver fibrosis in a model of primary 3D human liver spheroids. CTGF expression and production were increased upon treatment with TGF-β1, FFA or IL-6, suggesting a role of CTGF in ECM deposition during the development of liver fibrosis. Furthermore, treatment with recombinant CTGF showed that CTGF alone is capable of inducing a fibrosis-like phenotype in the spheroids and similar results were obtained by pharmacologically-induced CTGF expression using angiotensin II. We also found that silencing CTGF reduces both TGF-β1-induced and FFA-induced COL1A1 deposition in the spheroids. Taken together, our results indicate that CTGF and CTGF downstream signaling events are involved in the fibrosis-inducing processes triggered by FFA and TGF-β1. However, although CTGF silencing reduced COL1A1 deposition following FFA- or TGF-β1-induced fibrosis, the incomplete inhibition of COL1A1 formation also suggests the participation of CTGF-independent mechanisms. A schematic description of the proposed role and function of CTGF in liver fibrosis is presented in Figure 7.

We found that baseline CTGF expression in the spheroids was low, which is in line with tissue data from healthy livers [29,30]. TGF-β1 has previously been described to increase CTGF expression in hepatocytes, portal fibroblasts, cholangiocytes, biliary epithelial cells and hepatic stellate cells in vivo [4,9,31,32]. Treatment of the spheroids with TGF-β1 revealed increased ECM deposition, in line with data presented previously [19], as well as increased expression of cell stress markers, such as TGF-β1, LOX and α-SMA. In addition, the CTGF expression was highly induced by TGF-β1, both at the mRNA and protein levels. This process has previously been suggested to involve several signaling pathways, such as Smad [4,33], Erk [34], AKT [35] and Hippo pathways [36], causing increased ECM deposition [4,31]. The main hepatic cellular source of CTGF production has been described to be the stellate cells [12], which also represent the majority of cells within the NPC fraction used in this study. However, in the spheroid system, we observed a TGF-β1-dependent increase in CTGF production in both hepatocyte monocultures, as well as in the co-cultures. The latter indicates that PHH are capable of producing CTGF after being stimulated with TGF-β1, which is a very strong inducer of fibrosis and epithelial-to-mesenchymal transition [37]. PHH may revert to a more mesenchymal state after TGF-β1 treatment, as COL1A1 and TGF-β1 itself are also increased.

In our model, elevated concentrations of saturated and unsaturated FFA are used to simulate circulating FFA concentrations as a means by which the effect of Western diet on NASH and liver fibrosis can be studied [38,39]. In total we used 480 μM of FFA in the model which is still in the normal range of FFA in human serum [40]. During NASH and liver fibrosis, vitamin-A-storing HSC are activated into proliferative fibrogenic myofibroblasts [7], having increased production of reactive oxygen species [41], increased inflammatory and immune responses and increased ECM production [42]. FFA treatment of the spheroids caused a significant increase in CTGF production, accompanied by an increase in COL1A1 and TGF-β1 deposition. One of the suggested pathways involved in FFA-induced CTGF is the Hippo pathway. We here evaluated whether the Hippo pathway was affected upon FFA treatment by adding the YAP signaling inhibitor verteporfin and a YAP signaling activator XMU MP 1. It has been shown in mice models that yes-associated protein 1 (YAP) trans-locates into the nucleus while ECM is being increasingly produced, where it acts as a transcriptional co-regulator increasing CTGF transcription [43]. We observed an induction of CTGF production upon both FFA and XMU MP 1 treatment, and a decrease in CTGF production with verteporfin alone or in combination with FFA, but did not detect strong YAP relocation after FFA treatment which occurred after XMU MP1 treatment (unpublished observations). This indicates that FFA-induced CTGF induction is probably not mediated by the Hippo or YAP signaling in this model. Thus, the exact mechanism of CTGF-supported liver fibrosis is still ambiguous. Evidence has been presented for a role of CTGF-integrin interactions causing activation of signal transduction systems involving kinases, such as FAK, AKT and ERK, and a profibrotic action of CTGF influenced by the composition of the ECM and the relative amount of CTGF in the ECM [11]. The results in the spheroid system indicate that CTGF signaling is a part of the response to high fat load and thus significantly contributes to the observed liver fibrosis.

The spheroid model enables studies of gene-specific knockdowns, as well as investigating the effects of exogenously added proteins. Treatment of the spheroids with recombinant CTGF caused significantly increased deposition of COL1A1 and TGF-β1. In addition, recombinant CTGF induced CTGF production itself in the spheroids, indicating a positive feedback loop created by CTGF stimulation and later supported by the increased TGF-β1 production causing increased ECM production. CTGF treatment also increased *IL6* expression in the spheroids. Enhanced IL-6 production due to CTGF had previously been observed in human synovial fibroblasts, indicating that activation of the integrin-dependent signaling pathway significantly contributes to IL-6 elevation [44]. Increased IL-6 production indicates a pro-inflammatory response of the spheroids to CTGF treatment, showing a reasonably comprehensive representation of liver fibrosis

IL-6 is an important inflammatory mediator contributing to the activation of HSC, presumably by upregulating COL1A1 synthesis via the TGF-β1/Smad signaling pathway [45,46]. It is also an important fibrogenic pro-inflammatory cytokine [47], mainly produced in liver sinusoidal endothelial cells, Kupffer cells and other infiltrating immune cells, as well as by hepatocytes and HSC [48,49]. It has been stated that hepatocytes are IL-6-responsive because they possess the IL-6 receptor, while it is still unclear whether HSC can directly respond to IL-6 cis-signaling themselves [50,51]. We observed that IL-6 treatment slightly increased production of COL1A1, TGF-β1 and CTGF in the co-culture spheroids, but to a lesser extent compared to FFA or TGF-β1 stimuli. This indicates that IL-6 indirectly contributes to the profibrotic phenotype. In contrast to our findings, IL-6-decreased CTGF expression in a 2D PHH model of primary fresh isolated rat hepatocytes or cryo-preserved human hepatocytes in 2D [52] is indicative of a benefit of using more complex 3D human in vitro systems that more reliably capture the in vivo situation in humans for a more complete analysis of the mechanisms involved in inflammation and fibrosis.

The role of CTGF in the development of fibrosis was also studied by angiotensin II-induced CTGF expression. In LX-2 cells, angiotensin II was found to induce CTGF expression and ECM accumulation via a TGF-β1-independent mechanism [26]. Furthermore, olmesartan, an AT1 receptor antagonist, was shown to reduce bile duct ligation-induced liver fibrosis in rats [53]. In our human liver spheroid system, angiotensin II did induce CTGF at both the mRNA and protein levels and caused increased COL1A1 and TGF-β1 production, accompanied with elevated *LOX* and *IL6* expression. However, the mechanism of angiotensin II-induced CTGF production in the spheroids was not TGF-β1 independent, which prevented us from exactly disentangling the angiotensin II-induced CTGF effects from the TGF-β1 effects.

The actual contribution of CTGF to liver fibrosis remains ambiguous because it is, as mentioned, tightly linked to the TGF-β1 dependent pathways. An estimate of the direct inducing effect of CTGF on fibrosis relative to that of TGF-β1 proved to be difficult due to CTGF-induced TGF-β1 expression in the spheroids. CTGF silencing in liver fibrosis rodent models has shown to cause a decrease in the transcription of *TGFB1*, *ACTA2* and *COL1A1* [54,55,56]. However, by silencing the *CTGF* expression in spheroids, COL1A1 production upon TGF-β1 and FFA treatment was significantly reduced, thus indicating that CTGF and CTGF signaling contribute to ECM production.

There are some limitations to the study presented. The main PHH donor used in the model is heterozygous for the PNPLA3 I148M mutation, predisposing for liver fibrosis development. Furthermore, we observed a large inter-individual variation between donors, which, however, resembles the situation in vivo in the general population. Due to the mechanistic nature of this study, we mostly utilized one PHH-NPC combination with reproducible and well pronounced effects to show the role of CTGF in a human 3D in vitro system. An important issue in the studies of CTGF signaling and effects is the unavailable knowledge regarding its primary targets, hindering us from detangling the effect of CTGF from the effect of TGF-β1. Further studies on this matter are warranted. Nevertheless, 3D human liver spheroids represent a valuable model due to their ability for in vivo-like behavior, comprehensively recapitulating liver fibrosis, and as such, in terms of ECM deposition and inflammation, are a model that would be very useful for screening of chemicals and targets of importance for inhibition of NASH.

## 5. Conclusions

The results presented indicate a significant role of CTGF in advancement of liver fibrosis. The results do suggest that the quantitative contribution of CTGF to liver fibrosis in the spheroid model is smaller compared to TGF-β1. Nevertheless, a pharmacological intervention to modulate CTGF function for inhibition of liver fibrosis in vivo might be addressed in the future.

## Figures and Tables

**Table 1:** Characteristics of primary human hepatocyte and non-parenchymal cell donors.

Donor	PHH	NPC	Origin	Cat. nu.	Age	Sex	Ethnicity	*PNPLA3* rs738409	*PNPLA3* rs2294918
Donor 1	X		KLC	S1506T	47	Female	Caucasian	Het.	WT
Donor 2	X		Lonza	HUM190171	48	Female	Caucasian	Het.	Hom.
Donor 3		X	KLC	S1493	63	Male	Caucasian	WT	WT

WT—homozygous for the reference allele of the respective polymorphism, Het.—heterozygous for the respective polymorphism, Hom.—homozygous for the alternative allele of the respective polymorphism.

## Data Availability

The data presented in this study is all available in the manuscript and in the supplementary materials.

## Supplementary Materials

The following supporting information can be downloaded at: https://www.mdpi.com/article/10.3390/cells12020302/s1, Figure S1: Characterization of non-parenchymal cells and response to TGF-β1 treatment; Figure S2: Effect of recombinant CTGF on viability and functionality; Figure S3: Angiotensin II-induced CTGF expression and production; Table S1: TaqMan probes used for RT-PCR; Table S2: List of the used antibodies.
