# Supplementary material for: The Role of CTGF in Liver Fibrosis Induced in 3D Human Liver Spheroids
Source: Cells. 2023 Jan 13;12(2):302. doi: 10.3390/cells12020302 (PMC9857203; doi:10.3390/cells12020302)
Supplement: Supplementary file 1 [file cells-12-00302-s001.zip › cells-2134695-supplementary.pdf]

Supplementary table S1: TaqMan probes used for RT-PCR

Gene	TaqMan probe number	Company
COL1A1	Hs00164004_m1	Thermo Fisher
CTGF	Hs00170014_m1	Thermo Fisher
LOX	Hs00942483_m1	Thermo Fisher
TGFB1	Hs00998133_m1	Thermo Fisher
IL6	Hs00174131_m1	Thermo Fisher
TBP	Hs00427620_m1	Thermo Fisher

Supplementary table S2: List of the used antibodies

Primary Antibody	Species	Company	Cat. No.	Dilution
COL1A1	Rabbit	Abcam	ab34710	1:200
TGF-β1	Rabbit	Abcam	ab92486	1:200
α-SMA	Mouse	Abcam	ab7817	1:200
CTGF	Rabbit	Abcam	ab6992	1:300
Albumin	Mouse	Santa Cruz	sc-51515	1:200
CD36	Mouse	Abcam	ab17044	1:200
Fibronectin	Rabbit	Abcam	ab2413	1:200
Vimentin	Rabbit	Abcam	ab92547	1:200
CD32b	Rabbit	Abcam	ab151497	1:200
LYVE1	Rabbit	Abcam	ab281587	1:200
CD45	Mouse	Invitrogen	14-0459-82	1:200
CD31	Mouse	Abcam	ab9498	1:200
CD146	Mouse	Invitrogen	14-1469-82	1:200
vWF	Rabbit	Daco	A008229-2	1:200
CD163	Goat	R&D systems	AF1607	1:200
CD68	Mouse	Santa Cruz	sc-70761	1:200

Secondary Antibody	Company	Cat. No.	Dilution
Alexa Fluor 488 Goat-anti-Rabbit	Thermo Fisher	A-11008	1:500
Alexa Fluor 555 Donkey-anti-Rabbit	Thermo Fisher	A-31570	1:500
Alexa Fluor 555 Donkey-anti-Goat	Thermo Fisher	A-21432	1:500

Figure S1: Characterization of non-parenchymal cells and response to TGF-β1 treatment

A 2D non-parenchymal cells stained for different cell markers

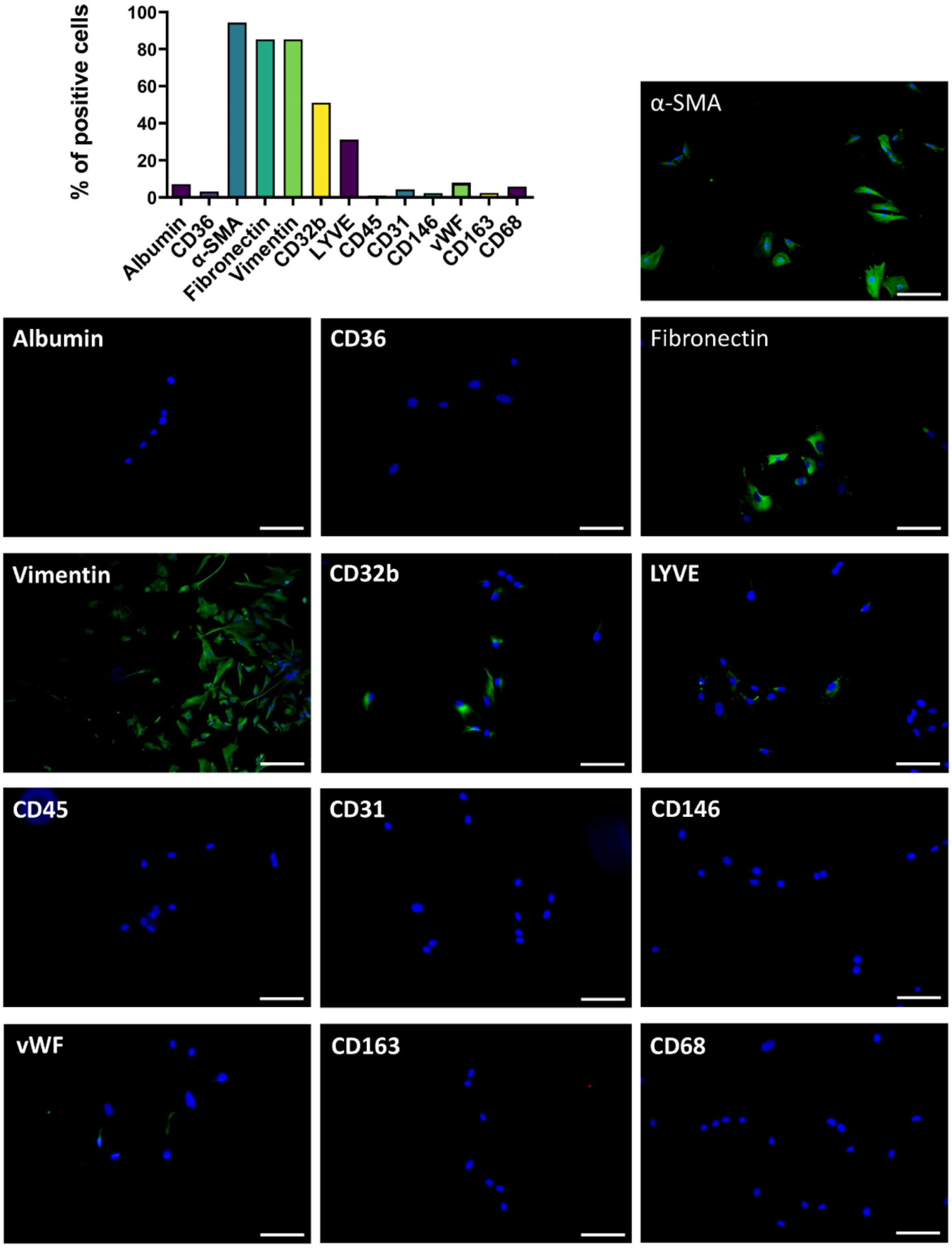

B **Variation between co-culture spheroids in response to TGF-β1 treatment within one experiment - COL1A1**

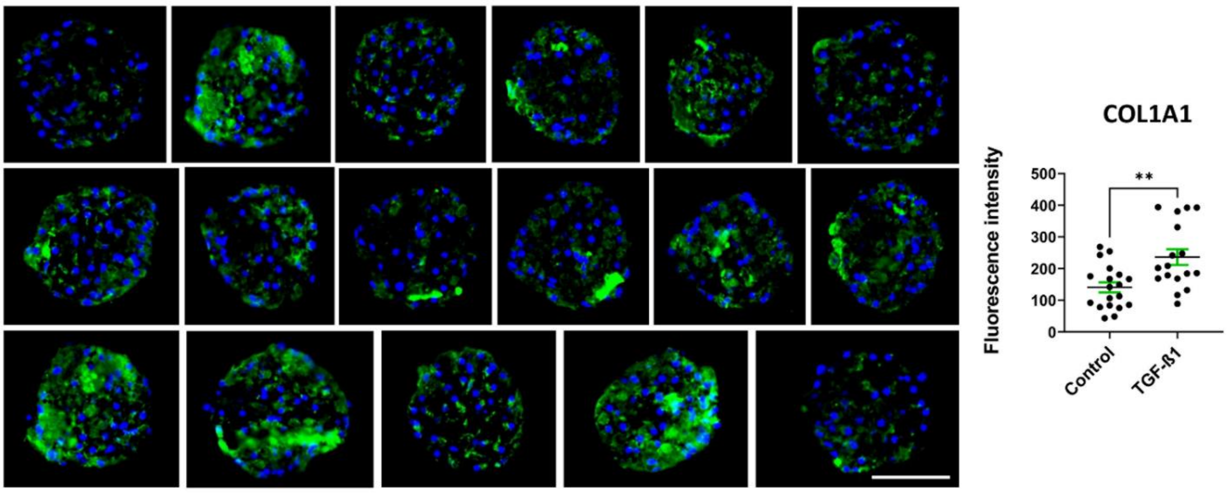

Variation between co-culture spheroids in response to TGF-β1 treatment within one experiment - CTGF

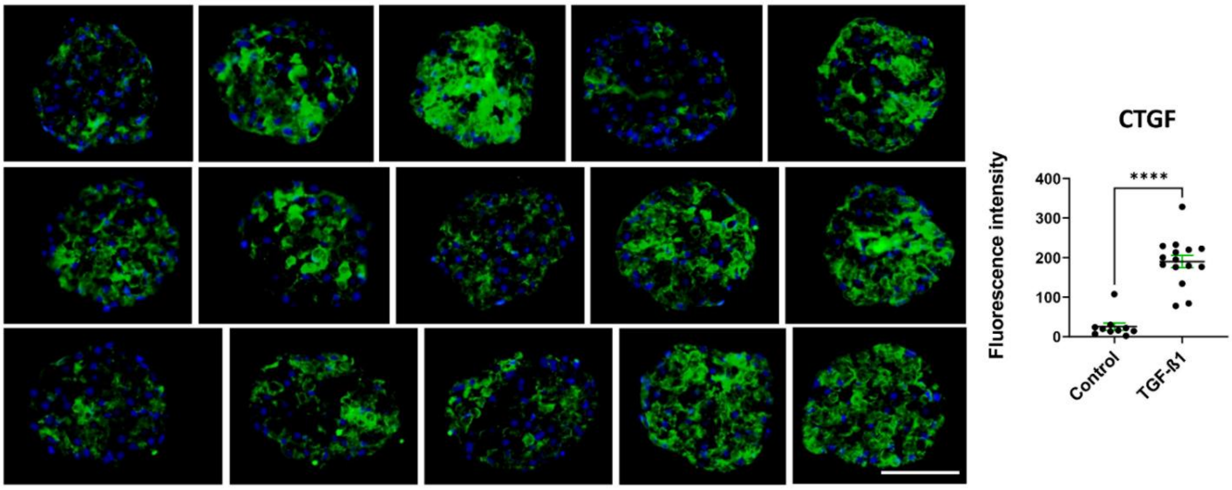

C **CTGF in cultured 2D primary human non-parenchymal cells and in freshly thawed 2D primary human hepatocytes**

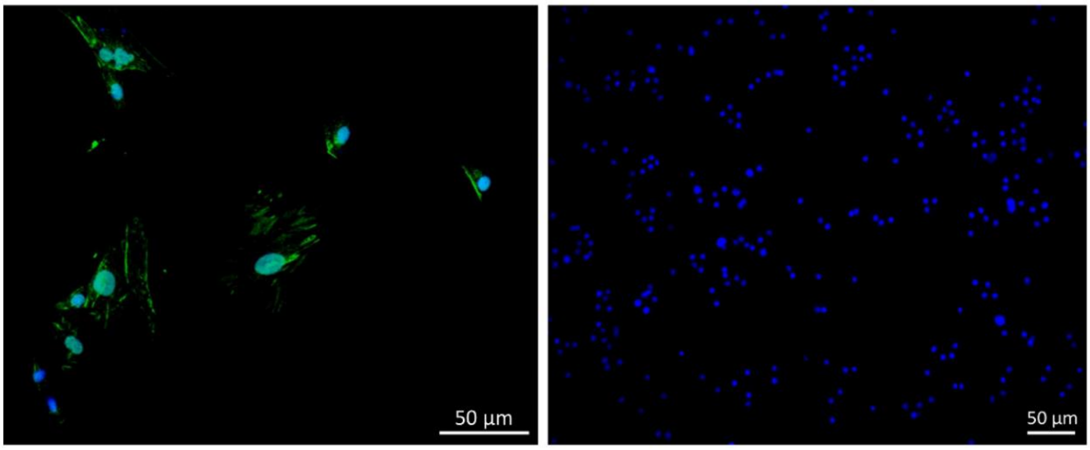

D **Deposition of COL1A1, TGF-β1, and α-SMA in mono-culture spheroids upon TGF-β1 treatment**

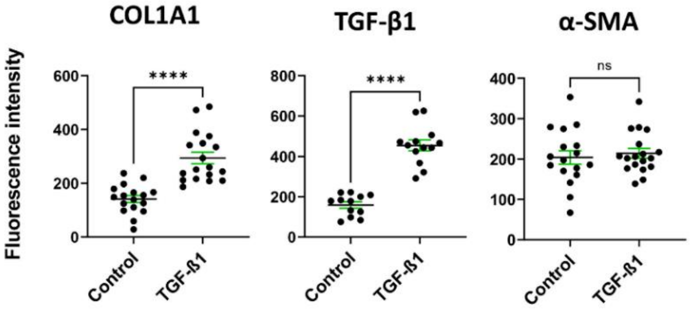

E **ATP measurements upon TGFβ1 treatment in the mono-culture and co-culture spheroids**

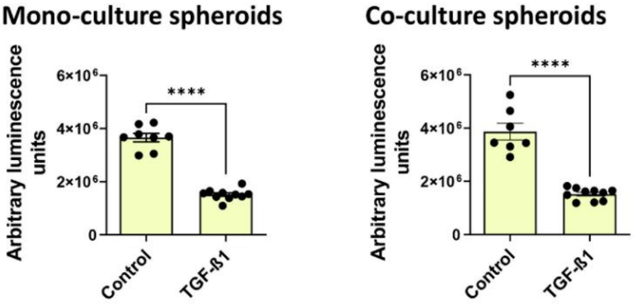

Figure S1: Characterization of non-parenchymal cells and TGF-β1 effects

(A) We characterized the non-parenchymal cells (NPC) used for the co-culture spheroids. After 2 days in culture in a 2D setting, cells were fixed and stained for several different cell type markers, such as hepatocyte markers (albumin, CD36), hepatic stellate cell (HSC) markers (α-SMA, fibronectin, vimentin), endothelial markers (CD31, CD32b, CD45, CD146, LYVE, vWF), and Kupffer cell markers (CD68, CD163). We observed that the greatest portion of cells was positive for α-SMA and the next highest percentage belonged to fibronectin and vimentin positive cells, suggesting that the majority of cells in the NPC fraction were HSC. Additionally, cells were also positive for the endothelial markers CD32b and LYVE, which shows presence of liver sinusoidal endothelial cells type 1 at the point of seeding. Other markers were very low or negative, which suggests that Kupffer cells and hepatocytes are not present in freshly thawed NPC. Bars in the plot represent the percentage of marker-positive cells. The size of the 2D cultured NPC is presented with a white scale bar of 100 µm.

(B) We show a great variability among spheroids in terms of response to chemical stimuli, in this case to TGF-β1 treatment with the consequential elevation in production of COL1A1 (upper panel) and CTGF (lower panel). Every image taken (minimum 10 per condition per experiment) is analysed and quantified. Every dot in the plot on the right represents the quantified fluorescence intensity (integrated density of the respective staining divided by the area of the DAPI staining) of 1 stained spheroid in 1 experiment. The average of all quantified spheroids per condition per experiment is presented in the immunohistochemistry plots throughout the article. The presented images belong to the TGF-β1 treated spheroids. In the plots we show the comparison between the control and treated spheroids. The size of the spheroids is marked with a white scale bar of 100 µm.

(C) CTGF staining in the 2D cells is presented. On the left side we show cells from the NPC fraction stained for CTGF after 2 days in culture. We hereby show that these cells can produce CTGF in culture. On the right side we present stained freshly thawed primary human hepatocytes (PHH), which did not show any positive CTGF staining. The size of the 2D cultured cells is presented with a white scale bar of 50 µm.

(D) Mono-culture spheroids have the ability to respond to TGF-β1 treatment. Production of COL1A1 and TGF-β1 was elevated, however production of α-SMA was not. Here we show results of 1 experiment with multiple spheroids presented individually (one dot is one spheroid).

(E) TGF-β1 lowered the spheroids' viability substantially, measured by the ATP production, after 3 days of treatment already. Every dot presents 1 spheroid. Spheroids presented in these plots originate from 3 separate experiments.

Nuclei in the immunohistochemistry images are shown in blue. Every dot in the plots B, E, and F present 1 spheroid. Data is shown as mean ± SEM. Statistical analysis – Student's t-test; ** $p < 0.01$, **** $p < 0.0001$.

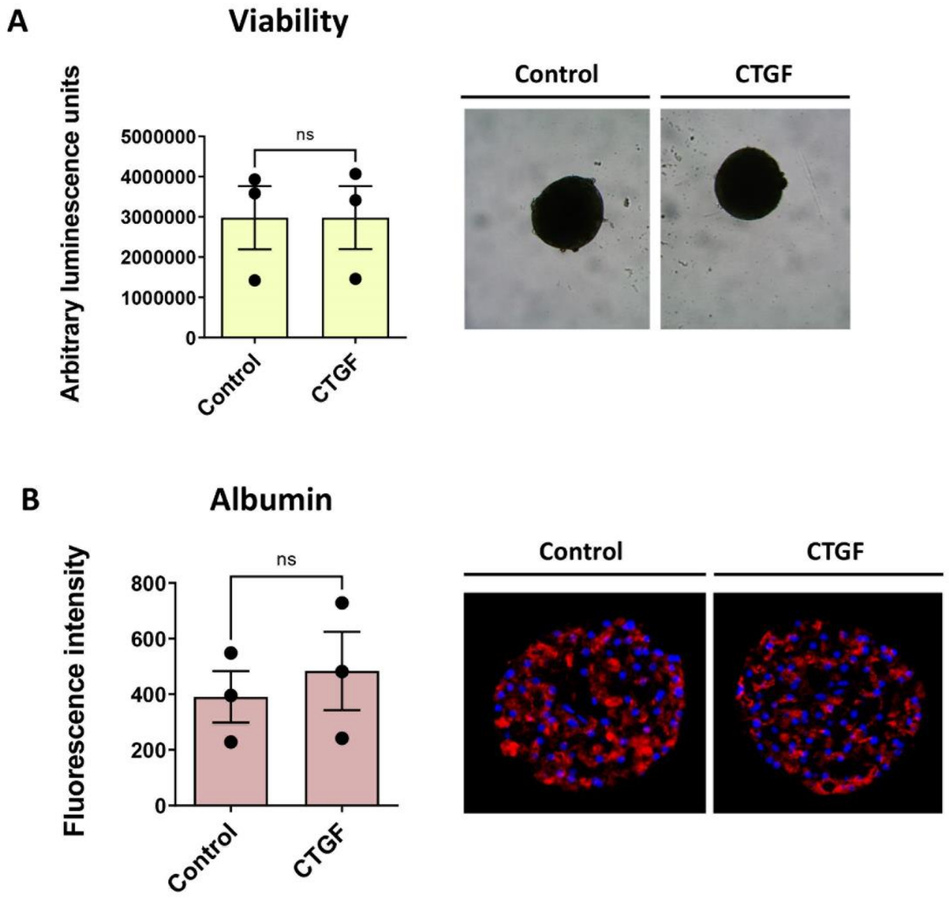

Figure S2: Effect of recombinant CTGF on viability and functionality

(A) Treatment of spheroids with recombinant CTGF for 7 days did not affect viability of spheroids, which was measured by quantification of the released ATP. One dot represents the average of the ATP measurements in at least 4 spheroids per condition per experiment. (n=3)

(B) Treatment of spheroids with recombinant CTGF also did not affect the functionality of hepatocytes in the spheroids, which was measured by the albumin staining. One dot represents the average of at least 10 imaged spheroids per condition per spheroid. (n=3)

Figure S3: Angiotensin II induced CTGF expression and production

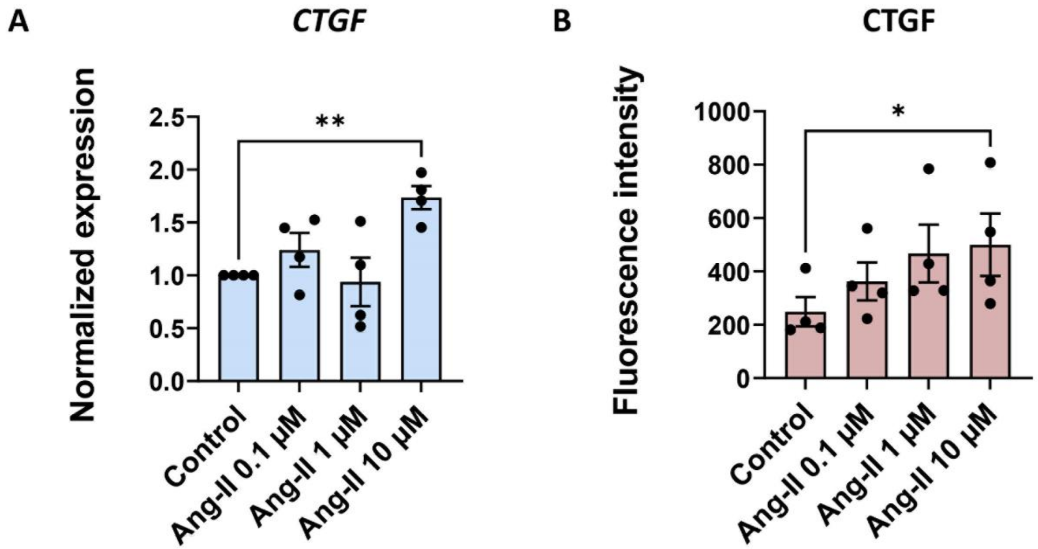

Angiotensin II induced CTGF expression and production in a concentration-dependent manner in co-culture spheroids

(A) *CTGF* expression was measured after 7 days of treatment with angiotensin II. We observed a significant elevation of *CTGF* expression with the highest concentration of angiotensin II, if compared to the respective control. The lower 2 concentrations did not affect the expression significantly. Every dot presents the average fold change in 1 experiment. (n=4)

(B) CTGF production was measured after 7 days of treatment with angiotensin II with immunohistochemistry. A concentration-dependent increase in CTGF production was observed, with a significant elevation observed with the highest concentration. Every dot represents an average fluorescence intensity of at least 10 different imaged spheroids per condition per experiment. (n=4)

Blue plots present the qPCR data, while the red plots present the immunohistochemistry data. Data is shown as mean ± SEM. Statistical analysis – Student's t-test; * < 0.05, ** p < 0.01.
